# Use of tiotropium in patients with COPD aged 80 years and older

**DOI:** 10.3892/etm.2013.956

**Published:** 2013-01-15

**Authors:** HIROAKI SATOH, KATSUNORI KAGOHASHI, GEN OHARA, SHINYA SATO, KUNIHIKO MIYAZAKI, KENSUKE NAKAZAWA, TOMOHIRO TAMURA, KOICHI KURISHIMA, MIO KAWAGUCHI, NOBUYUKI HIZAWA

**Affiliations:** 1 Division of Respiratory Medicine, Mito Medical Center, University of Tsukuba, Ibaraki, Japan; 2 Division of Respiratory Medicine, Institute of Clinical Medicine, University of Tsukuba, Ibaraki, Japan

**Keywords:** chronic obstructive pulmonary disease, tiotropium, elderly, octogenarian, comorbid disease

## Abstract

The purpose of this study was to examine clinical features and treatment modality approaches in patients with chronic obstructive pulmonary disease (COPD), particularly in those aged 80 years and older. Using databases available at Mito Kyodo General Hospital (Japan), the medical records of COPD patients between April 2009 and December 2011 were retrospectively reviewed. The patient population was divided into three age groups; less than 70 years (the <70 age group), between 70–79 years (the 70–79 age group) and 80 years or older (the ≥80 age group). Demographic data, as well as the efficacy and safety of tiotropium, were compared between the three groups. Patients in the ≥80 age group comprised 35.6% of the study population with COPD (n=174). The ≥80 and 70–79 age groups demonstrated a higher proportion of comorbid disease compared with the <70 age group. A subjective improvement of dyspnea on effort as well as no additional adverse effects were observed in the ≥80 age group, similar to the other two age groups. However, higher incidence of acute exacerbation of COPD in patients aged ≥80 years old was found, particularly in those with comorbid disease. The efficacy and safety of tiotropium in COPD patients in the ≥80 age group were almost identical to patients <80 years old, however, physicians must be cautious with acute exacerbation of COPD in the extremely elderly population with comorbid disease.

## Introduction

In developed countries, populations are becoming older with the incidence of respiratory diseases highest amongst the elderly (1,2). Among them, the incidence, morbidity and mortality of chronic obstructive pulmonary disease (COPD) has increased worldwide during the last few decades (1). Factors such as recent improvements in the medical management of COPD, the availability of new drugs and an improved standard of medical care with more widespread health services may help to provide favorable treatment outcomes. Tiotropium, an agent in a new class of anticholinergics, exhibits a longer duration of action due to prolonged muscarinic receptor antagonism (3,4). These effects have the therapeutic advantage of making once-daily dosing in patients possible (5).

The majority of diagnosed cases of COPD are those between the ages of 60 and 80 years, and as a result, there are few data relating to the clinical characteristics, disease status, treatment modality, efficacy and safety of tiotropium treatment in patients aged ≥80 years (6). Too few patients >80 years have been included in previous clinical trials to allow any significant conclusions with regard to this age group to be made. Therefore, additional studies specifically focusing on these elderly patients are required. This study reports data concerning the efficacy and safety of tiotropium in COPD patients, especially in those aged ≥80 years.

## Patients and methods

### Clinicopathological data

For all consecutive patients with COPD, clinicopathological data were obtained by retrospective review from the databases at the University of Tsukuba, Mito Medical Center (Mito Kyodo General Hospital, Japan). Patients were diagnosed functionally and treated with tiotropium (once daily via Respimat 5 *μ*l or HandiHaler^®^ 18 *μ*g for >4 weeks) between April 2009 and January 2012 at our hospital. Demographic data, including age, gender and comorbid disease, were retrieved from each patient’s medical record. This study was approved by the Institutional Ethics Committee of the Mito Kyodo General Hospital. The entire patient population was divided into three age groups; less than 70 years (the <70 age group), 70–79 years (the 70–79 age group) and 80 years or older (the ≥80 age group). Demographic data, as well as the efficacy and safety of tiotropium, were compared between the three age groups. The efficacy of tiotropium was evaluated at 1 month or more from the initiation of the inhalation therapy. Adverse effects were counted as they occurred during the study period. Acute exacerbation of COPD was defined according to the review by Effing *et al* (6). Written informed patient consent was obtained from each patient.

### Statistical analysis

Differences between the distribution of subpopulations between each group were analyzed using the Mann-Whitney U test, Kruskal-Wallis test and Chi-square test. Statistical significance between paired forced expiratory volume in 1 sec (FEV1) levels was evaluated by the Wilcoxon signed rank test. All statistical analyses were performed using SPSS 10.1 for Windows (SPSS, Inc., Chicaco, IL, USA) and P<0.05 was considered to indicate a statistically significant result.

## Results

### Patient characteristics

A total of 174 patients with COPD were treated with tiotropium during the study period. Among them, 62 patients (35.6%) were ≥80 years (median, 84 years), 59 patients (33.9%) were between 70–79 years (median, 75 years) and 53 patients (30.5%) were <70 years (median, 65 years; Table I). Males constituted 86.2% of the study population and the age distribution was comparable between males (≥80 years, 34.7%; 70–79 years, 33.3%; <70 years, 32.0%) and females (≥80 years, 41.7%; 70–79 years, 37.5%; <70 years, 20.8%; P=0.5386; Table I).

Forty-eight of the patients aged ≥80 years (77.4%) had one or more comorbid disease, compared with 72.9 and 49.1% of patients aged between 70–79 and <70 years old displaying one or more comorbid disease, respectively. There was a significant difference in the ratio of having one or more comorbid disease between those aged ≥70 years and those <70 years (P=0.0009). However, no significant difference in the ratio between those aged ≥80 years and those <80 years was observed (P= 0.5634). There was a significant difference in the ratio of having prostate disease between those aged ≥70 years and those <70 years (P=0.0395), however no significant difference in the ratio between those aged ≥80 years and those <80 years was observed (P=0.0709).

### Treatment of COPD

Treatment and outcome of COPD is shown in Table II. Inhaled corticosteroid/long-acting β2-agonist (ICS/LABA) was prescribed in 14 (22.6%) patients aged ≥80 years, 16 (27.1%) patients aged between 70–79 years and 24 (45.3%) patients <70 years. There was a statistically significant difference in the ratio between patients ≥70 years and those <70 years (P=0.0002). LABA was prescribed in 15 (24.2%) patients aged ≥80 years, 12 (20.3%) patients aged between 70–79 years and 1 (1.9%) patient <70 years. There was a statistically significant difference in the ratio between patients ≥70 years and those <70 years (P=0.0007).

### Efficacy of tiotropium

After 1 month or longer treatment with tiotropium inhalation, subjective improvement of dyspnea on effort was observed in 41 (66.1%) patients aged ≥80 years, 43 (72.9%) patients aged between 70–79 years and 41 (77.4%) patients <70 years. There was no statistically significant difference in the ratio between patients aged ≥80 years and those <80 years (P=0.2128).

Sixty-eight (39.1%) patients had a pulmonary function test (PFT) before the initiation of tiotropium treatment and FEV1 was 1.14 liters. Only 3 (4.8%) patients aged ≥80 years, 11 (18.6%) aged between 70–79 years and 19 (35.8%) patients <70 years had a respiratory function test prior to tiotropium therapy and 1–3 months after the initiation of the therapy. Due to the small number of patients, improvement of FEV1 was not observed in each group of patients (P=0.1088, P=0.0997 and P=0.2956, respectively; Wilcoxon signed rank test).

In the patient group aged ≥80 years, 17 (27.4%) patients had acute exacerbations of COPD, however, 3 (5.1%) and 5 (15.2%) patients had the exacerbations in the patient groups aged 70–79 years and <70 years, respectively. There was a significant difference in the incidence of the exacerbations between the most elderly group and the counter groups of patients (P=0.0003). In the patient group aged ≥80 years, 14 (82.4%) patients who experienced the exacerbation had one or more comorbid disease.

### Safety

Only one (0.6%) patient experienced faintness and nausea, respectively. These complaints were observed in patients aged between 70–79 years. However, these side-effects immediately disappeared after tiotropium inhalation treatment was terminated. During the study period, no patient experienced hoarseness, arrhythmia, glaucoma attack, urinary retention, dry mouth, constipation, gastrointestinal obstruction or dysuria. Therefore, an increased incidence of adverse effects was not observed in the ≥80 years age group.

## Discussion

There has been an increasing interest in the treatment of elderly patients with COPD (1,7,8). However, clinical information with regard to COPD patients aged ≥80 years has not been fully available since such patients are not usually included in clinical trials and retrospective care analysis. Therefore, a degree of scientific uncertainty regarding the risks and benefits of treatment with tiotropium in COPD patients aged ≥80 years currently exists. We retrospectively reviewed the clinical data from consecutive patients with COPD treated with tiotropium at our hospital to assess its efficacy and safety for treatment in the elderly population. Previous studies have reported the efficacy and safety of tiotropium therapy for COPD patients (9–11). In these studies, the mean age of the patients evaluated were 70.2 years (11), 68.2–69.2 years (9) and 65.2 years (10). In our study, the median age of all patients was 75 years, and 69.5% of them were ≥70 years old. It is noteworthy that the ≥80 age group accounted for 35.6% of all our COPD patients. As for FEV1 in previous studies, its mean value was 1.02 (9) and 1.17 liters (11). In our study, median FEV1 of patients was 1.14 liters. Thus, severity of pulmonary function in our patients may be compatible with those in previous studies (9,11).

Aging is associated with a higher prevalence of comorbid disease (12,13), particularly in those patients with a long history of smoking who are predisposed to comorbid diseases such as COPD and cardiovascular disease, as well as other smoking-related malignancies (14). The ≥80 age group had a higher proportion of patients with comorbid disease than those in septuagenarians and patients <70 years of age. Among them, however, there was a significant difference in the ratio of having prostate disease between those aged ≥70 years and those <70 years, but no difference was observed in the ratio between those aged ≥80 years and those <80 years.

In our study, achievement of subjective improvement in dyspnea on effort was equivalent in the ≥80 and <80 years patient age groups. However, there was a significant difference in the incidence of exacerbations between the ≥80 age group and the counter groups of patients. One of the reasons may be due to the severity of COPD itself as well as that of numerous other comorbid diseases in the elderly. Tiotropium inhalation is generally well tolerated in patients with COPD, without anticholinergic adverse events (e.g. dry mouth, constipation, gastrointestinal obstruction, dysuria) (15). Kesten *et al* reported that nasopharyngitis and dry mouth were two of the most common adverse events in tiotropium therapy (16). In our study, arrhythmia, glaucoma attack, urinary retention, dry mouth, constipation, gastrointestinal obstruction and nasopharyngitis were not observed, even in the ≥80 age group of patients.

Our results revealed some novel findings, but there were a number of limitations in this study. This was a small-sized retrospective study in one institution. PFT was not evaluated in every patient and quality of life analysis was not performed in this study. Therefore, we determined that conclusive outcomes were not able to be derived from this study. However, our methodology, which was based on an audit of information obtained from clinical practice documented in patient records, may provide some clinical information which otherwise cannot be observed from clinical trials. As numerous elderly patients had severe comorbid disease, they were as a result not suitable for PFT test, which requires their maxmum effort to perform. We believe that reporting the treatment experiences of COPD patients, including those elderly patients who cannot perform PFT due to complications, is of significance to clinical practice.

The efficacy and safety of tiotropium in COPD patients aged ≥80 years was almost identical to that of patients aged <80 years, however, physicians must be cautious with acute exacerbation of COPD in the extremely elderly population, particularly those with comorbid disease.

## Figures and Tables

**Table I:** Characteristics of 174 patients with chronic obstructive pulmonary disease.

Characteristic	Value
Age, n (%): median	
≥80 years	62 (35.6): 84
70–79 years	59 (33.9): 75
<70 years	53 (30.5): 65
Gender, n (%)	
Male	150 (86.2)
Female	24 (13.8)
Age of patients with comorbid disease, n (%)	
≥80 years	48 (77.4)
70–79 years	43 (72.9)
<70 years	26 (49.1)
Age of patients with prostate disease, n (%)	
≥80 years	7 (11.3)
70–79 years	14 (23.7)
<70 years	3 (5.7)

**Table II:** Characteristics of 174 patients with chronic obstructive pulmonary disease.

Variable	n (%)
Treatment	
LABA/ICS	
Aged ≥80 years old	14 (22.6)
Aged 70–79 years	16 (27.1)
Aged <70 years	24 (45.3)
LABA	
Aged ≥80 years old	15 (24.2)
Aged 70–79 years	12 (20.3)
Aged <70 years	1 (1.9)
Outcome	
Improvement of symptoms	
Aged ≥80 years old	41 (66.1)
Aged 70–79 years	43 (72.9)
Aged <70 years	41 (77.4)
Admission during the study period	
Aged ≥80 years old	17 (27.4)
Aged 70–79 years	3 (5.1)
Aged <70 years	5 (9.4)
Adverse effects	
Faintness	1 (0.6)
Nausea	1 (0.6)
Arrhythmia	0
Glaucoma attack	0
Urinary retention	0
Dry mouth	0

LABA, long-acting β-agonists; ICS, inhaled corticosteroids.

## References

[1] Mannino DM, Buist AS (2007). Global burden of COPD: risk factors, prevalence, and future trends. Lancet.

[2] van de Nadort C, Smeets HM, Bont J, Zuithoff NP, Hak E, Verheij TJ (2009). Prognosis of primary care patients aged 80 years and older with lower respiratory tract infection. Br J Gen Pract.

[3] Littner MR, Ilowite JS, Tashkin DP (2000). Long-acting broncho-dilation with once-daily dosing of tiotropium (Spiriva) in stable chronic obstructive pulmonary disease. Am J Respir Crit Care Med.

[4] Disse B, Speck GA, Rominger KL, Witek TJ, Hammer R (1999). Tiotropium (Spiriva): mechanistical considerations and clinical profile in obstructive lung disease. Life Sci.

[5] Witek TJ, Souhrada JF, Serby CW, Disse B, Spector SS (1999). Tiotropium (Ba679). Pharmacology and early clinical observations. Anticholinergics in the Upper and Lower Airways.

[6] Effing TW, Kerstjens HA, Monninkhof EM (2009). Definitions of exacerbations: does it really matter in clinical trials on COPD?. Chest.

[7] Nilsson JL, Haupt D, Krigsman K, Moen J (2009). Asthma/COPD drugs reflecting disease prevalence, patient adherence and persistence. Expert Rev Respir Med.

[8] Snow V, Lascher S, Mottur-Pilson C, Joint Expert Panel on Chronic Obstructive Pulmonary Disease of the American College of Chest Physicians and the American College of Physicians-American Society of Internal Medicine (2001). Evidence base for management of acute exacerbations of chronic obstructive pulmonary disease. Ann Intern Med.

[9] Casaburi R, Mahler DA, Jones PW (2002). A long-term evaluation of once-daily inhaled tiotropium in chronic obstructive pulmonary disease. Eur Respir J.

[10] Lange P, Andersen KK, Munch E (2009). Quality of COPD care in hospital outpatient clinics in Denmark: The KOLIBRI study. Respir Med.

[11] Ichinose M, Fujimoto T, Fukuchi Y (2010). Tiotropium 5microg via Respimat and 18microg via HandiHaler; efficacy and safety in Japanese COPD patients. Respir Med.

[12] Barnes PJ (2008). Future treatments for chronic obstructive pulmonary disease and its comorbidities. Proc Am Thorac Soc.

[13] Tan SL, Wood AM (2009). Chronic obstructive pulmonary disease and comorbidity: a review and consideration of pathophysiology. Panminerva Med.

[14] Corsonello A, Antonelli-Incalzi R, Pistelli R (2011). Comorbidities of chronic obstructive pulmonary disease. Curr Opin Pulm Med.

[15] Keating GM (2012). Tiotropium bromide inhalation powder: a review of its use in the management of chronic obstructive pulmonary disease. Drugs.

[16] Kesten S, Jara M, Wentworth C, Lanes S (2006). Pooled clinical trial analysis of tiotropium safety. Chest.

